# Cucurbitacin B Down-Regulates TNF Receptor 1 Expression and Inhibits the TNF-α-Dependent Nuclear Factor κB Signaling Pathway in Human Lung Adenocarcinoma A549 Cells

**DOI:** 10.3390/ijms23137130

**Published:** 2022-06-27

**Authors:** Eiichi Kusagawa, Chiharu Okuda, Rikako Yamaguchi, Kaori Nakano, Yasunobu Miyake, Takao Kataoka

**Affiliations:** 1 Department of Applied Biology, Kyoto Institute of Technology, Matsugasaki, Sakyo-ku, Kyoto 606-8585, Japan; ksgw205@gmail.com (E.K.); chiharokuda@gmail.com (C.O.); ryamaguchi938@gmail.com (R.Y.); n.n.ka1025@gmail.com (K.N.); 2 Division of Molecular and Cellular Immunoscience, Department of Biomolecular Sciences, Faculty of Medicine, Saga University, Saga 849-8501, Japan; ymiyake@cc.saga-u.ac.jp

**Keywords:** cucurbitacin B, nuclear factor κB, TNF receptor 1

## Abstract

Pro-inflammatory cytokines, such as tumor necrosis factor-α (TNF-α), induce the expression of intracellular adhesion molecule-1 (ICAM-1) by activating the nuclear factor κB (NF-κB) signaling pathway. In the present study, we found that cucurbitacin B decreased the expression of ICAM-1 in human lung adenocarcinoma A549 cells stimulated with TNF-α or interleukin-1α. We further investigated the mechanisms by which cucurbitacin B down-regulates TNF-α-induced ICAM-1 expression. Cucurbitacin B inhibited the nuclear translocation of the NF-κB subunit RelA and the phosphorylation of IκBα in A549 cells stimulated with TNF-α. Cucurbitacin B selectively down-regulated the expression of TNF receptor 1 (TNF-R1) without affecting three adaptor proteins (i.e., TRADD, RIPK1, and TRAF2). The TNF-α-converting enzyme inhibitor suppressed the down-regulation of TNF-R1 expression by cucurbitacin B. Glutathione, *N*-acetyl-L-cysteine, and, to a lesser extent, L-cysteine attenuated the inhibitory effects of cucurbitacin B on the TNF-α-induced expression of ICAM-1, suggesting that an *α,β*-unsaturated carbonyl moiety is essential for anti-inflammatory activity. The present results revealed that cucurbitacin B down-regulated the expression of TNF-R1 at the initial step in the TNF-α-dependent NF-κB signaling pathway.

## 1. Introduction

Pro-inflammatory cytokines, such as those belonging to the tumor necrosis factor (TNF) family and interleukin-1 (IL-1) family, are mainly produced by macrophages [1]. In the intracellular signaling pathways, TNF-α and IL-1 primarily activate the transcription factor nuclear factor κB (NF-κB) [2,3]. NF-κB induces the transcriptional activation of many genes, some of which encode cell adhesion molecules, cytokines, and chemokines [4]. In vascular endothelial cells, cell adhesion molecules are up-regulated during inflammation and mediate the capture and migration of circulating leukocytes to local inflamed sites [5,6]. Intercellular adhesion molecule-1 (ICAM-1) is a cell adhesion molecule that regulates inflammatory and immune responses [7]. Its expression is mainly up-regulated at the transcriptional level by NF-κB subunits [8].

TNF-α binds to TNF receptor 1 (TNF-R1), which is widely expressed, and TNF receptor 2 (TNF-R2), the expression of which is more limited [9]. TNF-R1 possesses an extracellular cysteine-rich domain (CRD) that is essential for ligand binding and an intracellular death domain (DD) that mediates NF-κB and cell death signaling [10,11]. Upon binding with TNF-α, TNF-R1 recruits TNF receptor-associated death domain (TRADD), receptor-interacting protein kinase 1 (RIPK1), and TNF receptor-associated factor 2 (TRAF2) to its DD [3,9]. The TNF-R1 complex activates the inhibitor of NF-κB (IκB) kinase, which induces IκBα phosphorylation and its subsequent ubiquitination [12,13]. NF-κB heterodimers, such as those comprising RelA/p65 and p50, are sequestered to the cytosol by the association with IκBα, and proteasome-dependent degradation allows for the liberation of NF-κB heterodimers and their nuclear translocation [12,13].

**Figure 1:**
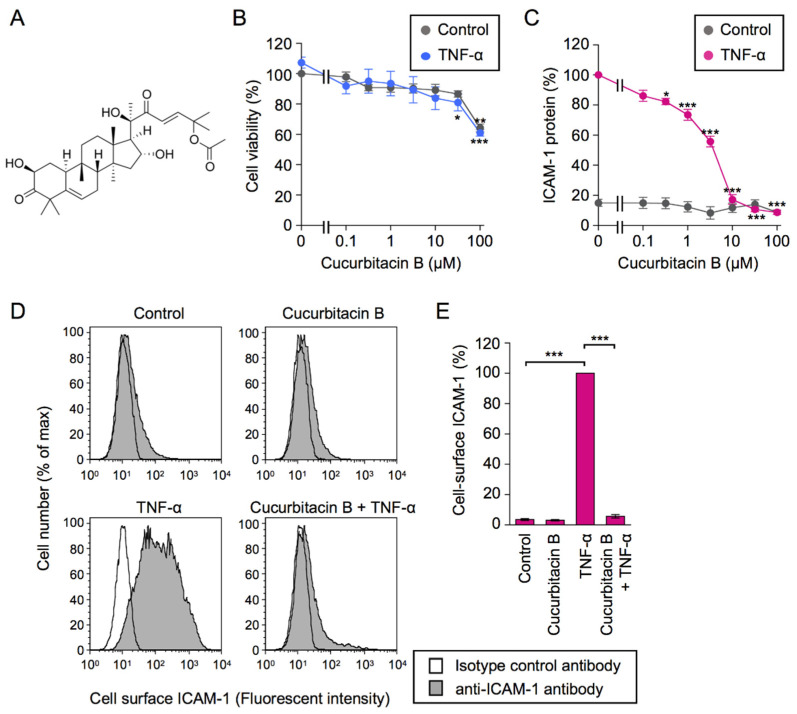
Cucurbitacin B down-regulated TNF-α-induced cell-surface ICAM-1 expression. (**A**) Structure of cucurbitacin B. (**B**,**C**) A549 cells were pretreated with serial dilutions of cucurbitacin B for 1 h and were then treated with (blue circles in (**B**) and red circles in (**C**)) or without (gray circles) TNF-α (2.5 ng/mL) in the presence of cucurbitacin B at the indicated final concentrations for 6 h. Cell viability (%) (**B**) and ICAM-1 protein (%) (**C**) are shown as the mean ± S.E. of three independent experiments. (**D**,**E**) A549 cells were incubated with cucurbitacin B for 1 h and then treated with or without TNF-α (2.5 ng/mL) in the presence or absence of cucurbitacin B (30 µM) for 6 h. Histograms are representative of three independent experiments (**D**). Cell-surface ICAM-1 (%) is shown as the mean ± S.E. of three independent experiments (**E**). * *p* < 0.05, ** *p* < 0.01, and *** *p* < 0.001.

Cucurbitacins, including cucurbitacin B (Figure 1A), belong to a group of triterpenes that are characterized by the cucurbitane skeleton (9*β*-methyl-19-norlanosta-5-ene) and are present in Cucurbitaceae and other plant families [14,15]. Cucurbitacins exert various biological effects, such as anti-inflammatory and anticancer activities [14,15,16,17,18]. The anti-inflammatory and anticancer activities of cucurbitacins are attributed at least partly to their inhibition of the constitutive and stimulation-induced NF-κB-dependent pathway [19,20,21,22,23,24,25,26,27,28,29,30,31,32,33,34]. Cucurbitacin B was shown to down-regulate the TNF-α-induced expression of NF-κB target genes and inhibit the transactivation activity of RelA without suppressing its nuclear translocation in human cervical carcinoma HeLa cells [21]. Cucurbitacins B and D reduced the TNF-α-induced expression of NF-κB reporter in human embryonic kidney 293 cells [34]. Cucurbitacin E suppressed the TNF-α-induced phosphorylation and nuclear translocation of RelA in human synoviocyte MH7A cells [26]. Collectively, these findings indicate that cucurbitacins inhibit the TNF-α-dependent NF-κB signaling pathway; however, the underlying mechanisms have not yet been elucidated.

We identified a number of natural and synthetic compounds that exhibited anti-inflammatory activity in evaluations of the down-regulation of ICAM-1 expression by pro-inflammatory cytokines [35]. In the present study, we found that cucurbitacin B reduced the level of ICAM-1 expression induced by TNF-α and IL-1α in human lung adenocarcinoma A549 cells. We further investigated the mechanisms by which cucurbitacin B down-regulates TNF-α-induced ICAM-1 expression. The results obtained revealed that cucurbitacin B down-regulated the expression of TNF-R1 at the initial step in the TNF-α-induced NF-κB signaling pathway.

## 2. Results

### 2.1. Cucurbitacin B Down-Regulated TNF-α-Induced ICAM-1 Expression

A549 cells were treated with serial dilutions of cucurbitacin B for 1 h and were then incubated with or without TNF-α or IL-1α for 6 h. Cell viability was assessed using the 3-(4,5-dimethylthiazol-2-yl)-2,5-diphenyltetrazolium bromide (MTT) assay. Cucurbitacin B at concentrations up to 30 µM did not markedly affect cell viability; however, a reduction in cell viability was observed at 100 µM (Figure 1B; Figure S1). TNF-α-induced expression of ICAM-1 protein was reduced by cucurbitacin B in a dose-dependent manner (Figure 1C). IL-1α-induced expression of the ICAM-1 protein was also decreased by cucurbitacin B (Figure S2). Flow cytometry confirmed that cucurbitacin B at 30 µM decreased the expression of cell-surface ICAM-1 induced by TNF-α and IL-1α (Figure 1D,E; Figure S3). Based on cell-ELISA, cucurbitacin B appeared to preferentially reduce the TNF-α-induced expression of ICAM-1 protein over that induced by IL-1α (Figure 1C; Figure S2). Therefore, we examined the mechanisms by which cucurbitacin B down-regulated TNF-α-induced ICAM-1 expression as well as the upstream intracellular process.

**Figure 2:**
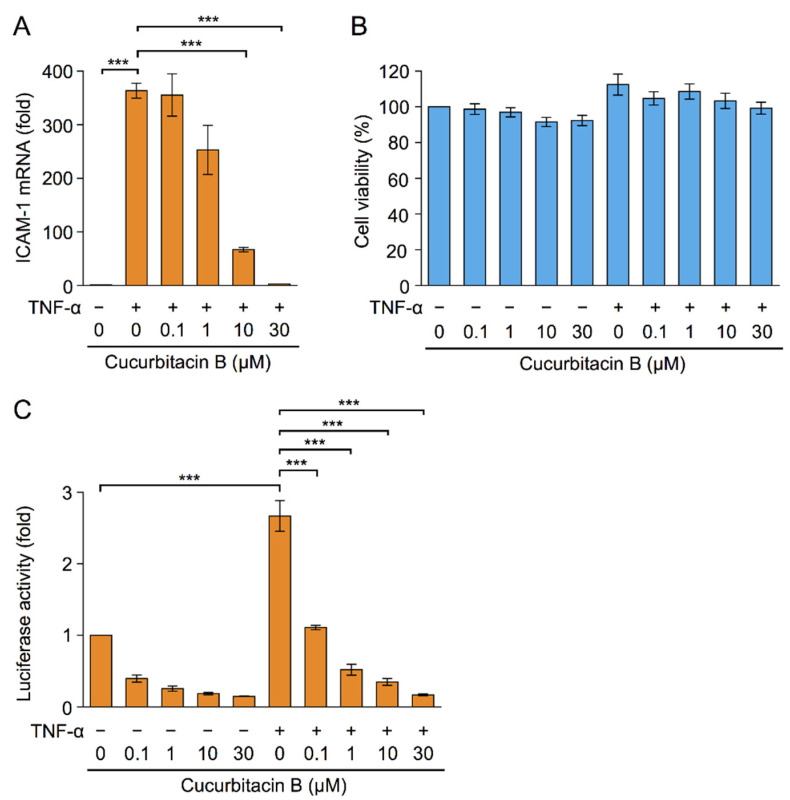
Cucurbitacin B inhibited TNF-α-induced ICAM-1 transcription. (**A**,**B**) A549 cells were pretreated with or without cucurbitacin B for 1 h and were then treated with (+) or (−) TNF-α (2.5 ng/mL) for 3 h. ICAM-1 mRNA (fold) is shown as the mean ± S.E. of three independent experiments (**A**). Cell viability (%) is shown as the mean ± S.E. of four independent experiments (**B**). (**C**) A549 cells were transfected with plasmid vectors encoding the ICAM-1 promoter-driven firefly luciferase reporter and cytomegalovirus (CMV) promoter-driven *Renilla* luciferase reporter for 17 h. Transfected A549 cells were pretreated with or without cucurbitacin B for 1 h and were then treated with (+) or without (−) TNF-α (2.5 ng/mL) for 3 h. ICAM-1 promoter-driven luciferase activity was normalized to CMV promoter-driven luciferase activity. Luciferase activity (fold) is shown as the mean ± S.E. of three independent experiments. *** *p* < 0.001.

ICAM-1 expression was up-regulated by TNF-α mainly at the transcriptional level [8]. In A549 cells, TNF-α increased the expression of ICAM-1 mRNA by more than 300-fold 3 h after the stimulation (Figure 2A). Cucurbitacin B reduced the TNF-α-induced expression of ICAM-1 mRNA (Figure 2A). Under these conditions, cucurbitacin B did not affect cell viability (Figure 2B). The luciferase reporter assay showed that cucurbitacin B inhibited TNF-α-induced ICAM-1 promoter-driven luciferase activity (Figure 2C). These results indicate that cucurbitacin B suppressed TNF-α-induced ICAM-1 transcription.

### 2.2. Cucurbitacin B Inhibited the TNF-α-Dependent NF-κB Signaling Pathway

**Figure 3:**
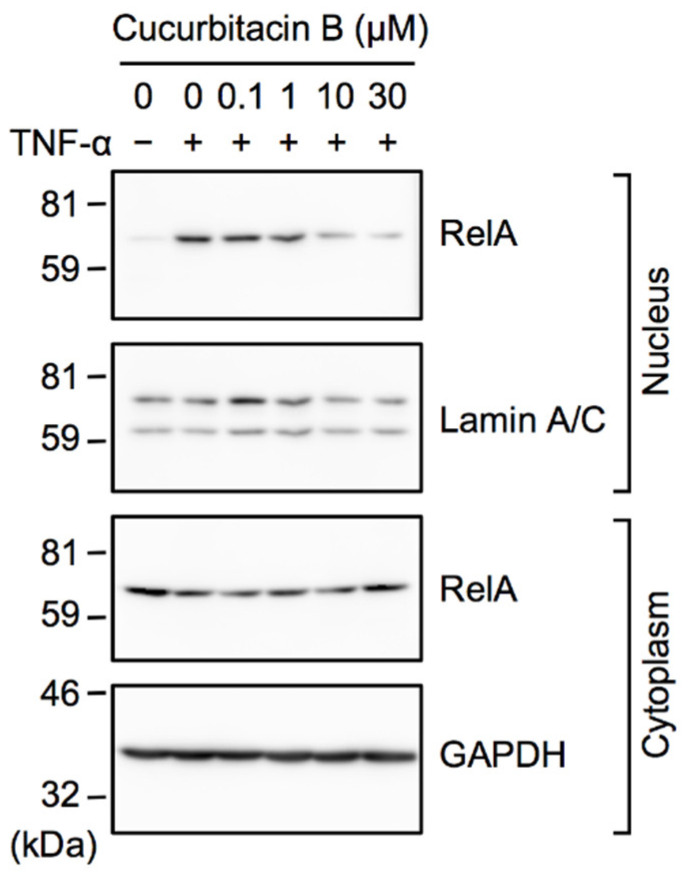
Cucurbitacin B inhibited the TNF-α-induced nuclear translocation of RelA. A549 cells were pretreated with or without cucurbitacin B for 1 h and were then treated with (+) or without (−) TNF-α (2.5 ng/mL) for 30 min in the presence or absence of cucurbitacin B at the indicated final concentrations, followed by the preparation of nuclear and cytoplasmic fractions. Western blots are representative of two independent experiments.

Upon TNF-α stimulation, the NF-κB subunit RelA translocated from the cytosol to the nucleus and bound to the ICAM-1 promoter in A549 cells [36]. A549 cells were pretreated with cucurbitacin B for 1 h and were then stimulated with TNF-α for 30 min. Cell lysates were separated into nuclear and cytoplasmic fractions. The amount of RelA in the nuclear fraction was markedly increased by TNF-α stimulation but decreased by cucurbitacin B (Figure 3). The amount of RelA in the cytoplasmic fraction was not markedly affected by cucurbitacin B (Figure 3).

**Figure 4:**
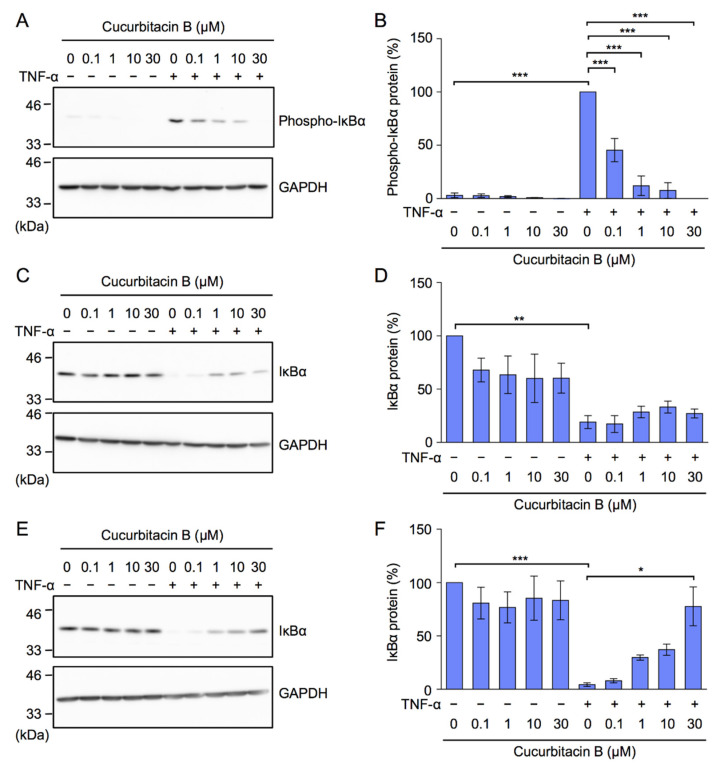
Cucurbitacin B inhibited TNF-α-induced IκBα phosphorylation and its subsequent degradation. (**A**–**F**) A549 cells were pretreated with or without cucurbitacin B for 1 h and were then treated with (+) or without (−) TNF-α (2.5 ng/mL) for 5 min (**A**–**D**) and 15 min (**E**,**F**) in the presence or absence of cucurbitacin B at the indicated final concentrations, followed by the preparation of cytoplasmic fractions. Western blots are representative of three independent experiments (**A**,**C**,**F**). The amounts of phospho-IκBα and IκBα were normalized to that of GAPDH. Phospho-IκBα protein (%) and IκBα protein (%) are shown as the mean ± S.E. of three independent experiments (**B**,**D**,**F**). * *p* < 0.05, ** *p* < 0.01 and *** *p* < 0.001.

In the TNF-α-induced NF-κB signaling pathway, a prerequisite to the nuclear translocation of RelA, we previously showed that IκBα was phosphorylated in A549 cells within 5 min and then degraded to undetectable levels after 15 min [37]. Cucurbitacin B inhibited the phosphorylation of IκBα in a dose-dependent manner (Figure 4A,B), and this was accompanied by a slight increase in the IκBα protein (Figure 4C,D). The degradation of IκBα 15 min after the TNF-α stimulation was inhibited by cucurbitacin B (Figure 4E,F). These results indicate that cucurbitacin B inhibited the TNF-α-dependent NF-κB signaling pathway.

### 2.3. Cucurbitacin B Down-Regulated the Expression of TNF-R1

**Figure 5:**
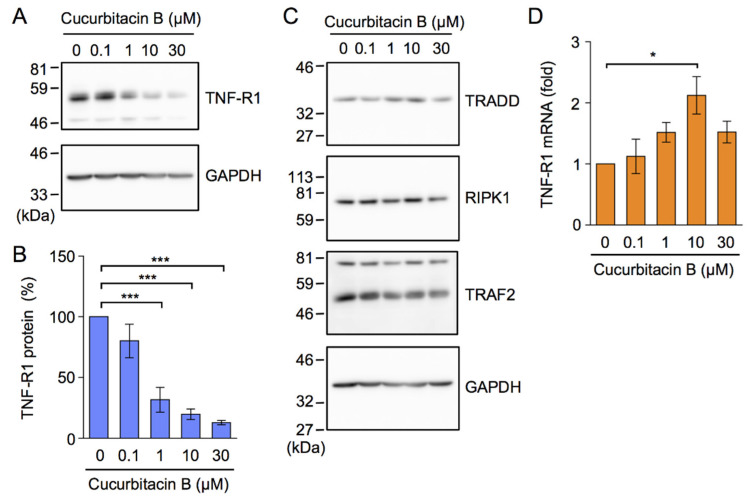
Cucurbitacin B down-regulated the expression of TNF-R1 without affecting the expression of TRADD, RIPK1, or TRAF2. (**A**–**C**) A549 cells were treated with or without cucurbitacin B at the indicated concentrations for 1 h, followed by the preparation of cytoplasmic fractions. TNF-R1 was detected by the anti-TNF-R1 antibody C25C1, which recognized an epitope in the intracellular domain. Blots are representative of three (**A**) and two (**C**) independent experiments. The amount of TNF-R1 was normalized to that of GAPDH. TNF-R1 protein (%) is shown as the mean ± S.E. of three independent experiments (**B**). (**D**) A549 cells were pretreated with or without cucurbitacin B at the indicated concentrations for 1 h. TNF-R1 mRNA (fold) is shown as the mean ± S.E. of three independent experiments. * *p* < 0.05 and *** *p* < 0.001.

We previously showed that A549 cells express TNF-R1 but not TNF-R2 [38]. Upon a stimulation with TNF-α, TNF-R1 recruits TRADD, RIPK1, and TRAF2 as major adaptor proteins, which is essential for the activation of IκB kinase [3,9]. To clarify the mechanisms by which cucurbitacin B inhibited the TNF-α-induced NF-κB signaling pathway, we measured the expression levels of TNF-R1 and adaptor proteins. Western blotting using an antibody recognizing TNF-R1 CRD showed that cucurbitacin B down-regulated the expression of TNF-R1 (Figure S4). Western blotting using another antibody recognizing an intracellular domain in TNF-R1 confirmed that cucurbitacin B down-regulated the expression of TNF-R1 (Figure 5A,B). In contrast, cucurbitacin B at concentrations up to 30 µM did not markedly down-regulate the expression of TRADD, RIPK1, or TRAF2 (Figure 5C). Cucurbitacin B did not reduce the expression of TNF-R1 mRNA; it actually increased its expression, particularly at 10 µM (Figure 5D).

**Figure 6:**
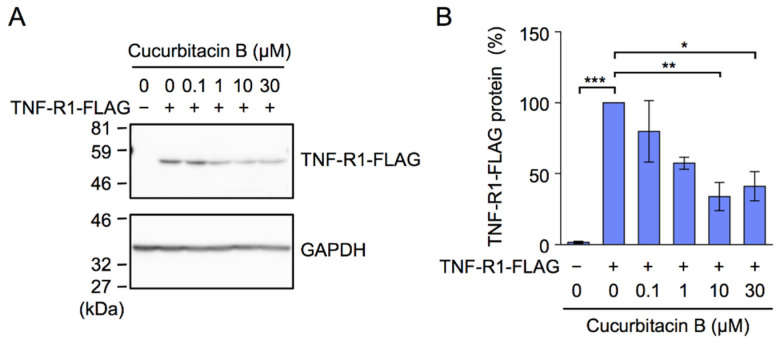
Cucurbitacin B down-regulated the expression of transfected TNF-R1. (**A**,**B**) A549 cells were transfected with a plasmid vector encoding FLAG-tagged human TNF-R1 for 18 h. Transfected A549 cells were treated with or without cucurbitacin B at the indicated concentrations for 1 h, followed by the preparation of cytoplasmic fractions. Blots are representative of three independent experiments (**A**). The amount of TNF-R1-FLAG was normalized to that of GAPDH. TNF-R1-FLAG protein (%) is shown as mean ± S.E. of three independent experiments (**B**). * *p* < 0.05, ** *p* < 0.01, and *** *p* < 0.001.

To clarify whether cucurbitacin B affected exogenous human TNF-R1, A549 cells were transiently transfected with an expression vector encoding C-terminal FLAG-tagged TNF-R1, driven by the constitutive CMV promoter, and were then treated with cucurbitacin B for 1 h. Western blotting using the anti-FLAG-antibody showed that cucurbitacin B decreased the amount of FLAG-tagged TNF-R1 (Figure 6A,B). These results indicate that cucurbitacin B selectively down-regulated the expression of TNF-R1 without affecting that of TRADD, RIPK1, or TRAF2.

### 2.4. Cucurbitacin B Promoted the TNF-α-Converting Enzyme (TACE)-Dependent Down-Regulation of TNF-R1

**Figure 7:**
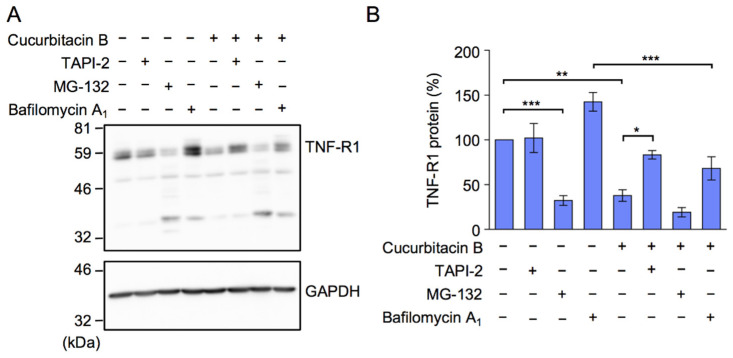
The TACE inhibitor TAPI-2 suppressed reductions in TNF-R1 protein levels by cucurbitacin B. (**A**,**B**) A549 cells were pretreated with (+) or without (−) bafilomycin A_1_, TAPI-2, or MG-132 for 1 h and then with (+) or without (−) cucurbitacin B (10 µM) for 1 h in the presence of bafilomycin A_1_ (100 nM), TAPI-2 (25 µM), or MG-132 (20 µM), followed by the preparation of whole cell lysates. TNF-R1 was detected by the anti-TNF-R1 antibody C25C1. Blots are representative of four independent experiments. The amount of TNF-R1 was normalized to that of GAPDH. TNF-R1 protein (%) is shown as the mean ± S.E. of four independent experiments. * *p* < 0.05, ** *p* < 0.01, and *** *p* < 0.001.

To investigate whether cucurbitacin B promoted TNF-R1 protein degradation, A549 cells were pretreated with the TACE inhibitor TAPI-2, the proteasome inhibitor MG-132, and the vacuolar type H^+^-ATPase inhibitor bafilomycin A_1_, which prevents lysosomal degradation, prior to the treatment with cucurbitacin B. TAPI-2 suppressed reductions in TNF-R1 protein levels in cucurbitacin B-treated A549 cells (Figure 7A,B). In contrast, cucurbitacin B still decreased TNF-R1 protein levels in the presence of bafilomycin A_1_ (Figure 7A,B). Unexpectedly, MG-132 alone decreased TNF-R1 protein levels, and this was accompanied by the appearance of smaller TNF-R1 fragments (Figure 7A,B). We previously demonstrated that MG-132 (20 µM) did not markedly affect the amount of RelA and IκBα in A549 cells during a 75- or 90-min incubation [36,39,40,41]. Although further studies are needed to clarify the mode of action of MG-132 on the degradation of TNF-R1, these results showed that cucurbitacin B promoted the TACE-dependent down-regulation of TNF-R1.

### 2.5. An α,β-Unsaturated Carbonyl Moiety Was Necessary for the Inhibitory Effect of Cucurbitacin B

**Figure 8:**
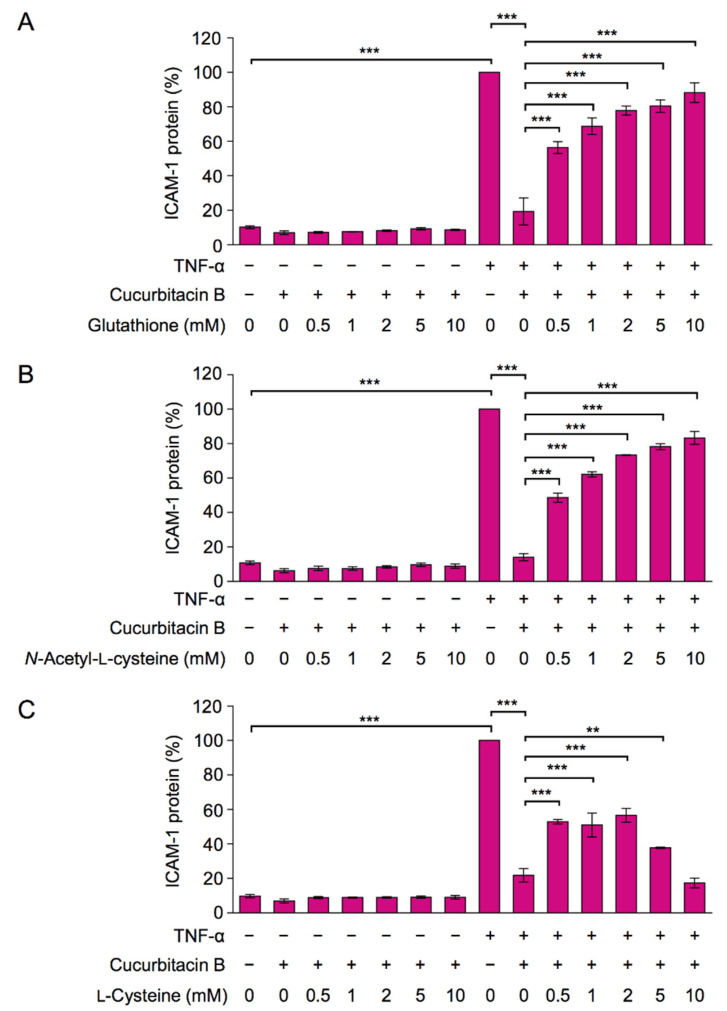
The inhibitory effect of cucurbitacin B was attenuated by glutathione, *N*-acetyl-L-cysteine, and, to a lesser extent, L-cysteine. (**A**–**C**) A549 cells were pretreated with serial dilutions of glutathione (**A**), *N*-acetyl-L-cysteine (**B**), and L-cysteine (**C**) for 1 h and were then treated with (+) or without (−) cucurbitacin B (10 µM) for 1 h and with (+) or without (−) TNF-α (2.5 ng/mL) for 6 h in the presence or absence of compounds at the indicated final concentrations. Cell-surface ICAM-1 (%) is shown as the mean ± S.E. of three independent experiments. ** *p* < 0.01 and *** *p* < 0.001.

Cucurbitacin B possesses an *α,β*-unsaturated carbonyl moiety (Figure 1A), which binds to thiol groups by the Michael reaction. To establish whether the *α,β*-unsaturated carbonyl moiety is essential for the inhibitory effects of cucurbitacin B, A549 cells were pretreated with thiol compounds and then with cucurbitacin B, followed by a TNF-α stimulation to induce ICAM-1 expression. Glutathione and *N*-acetyl-L-cysteine efficiently attenuated the inhibitory effects of cucurbitacin B on TNF-α-induced ICAM-1 expression (Figure 8A,B). The inhibitory effects of cucurbitacin B were also reversed, but to a lesser extent, by L-cysteine (Figure 8C).

Glutathione and *N*-acetyl-L-cysteine are often used as thiol-type antioxidants, while ascorbic acid and Trolox are antioxidants that lack thiol groups. Ascorbic acid and Trolox at concentrations up to 10 mM did not attenuate the inhibitory effects of cucurbitacin B on TNF-α-induced ICAM-1 expression (Figure S5). These results suggest that the *α,β*-unsaturated carbonyl moiety was necessary for the inhibitory effects of cucurbitacin B.

### 2.6. Cytochalasin D and Jasplakinolide Only Partially Down-Regulated TNF-α-Induced ICAM-1 Expression

**Figure 9:**
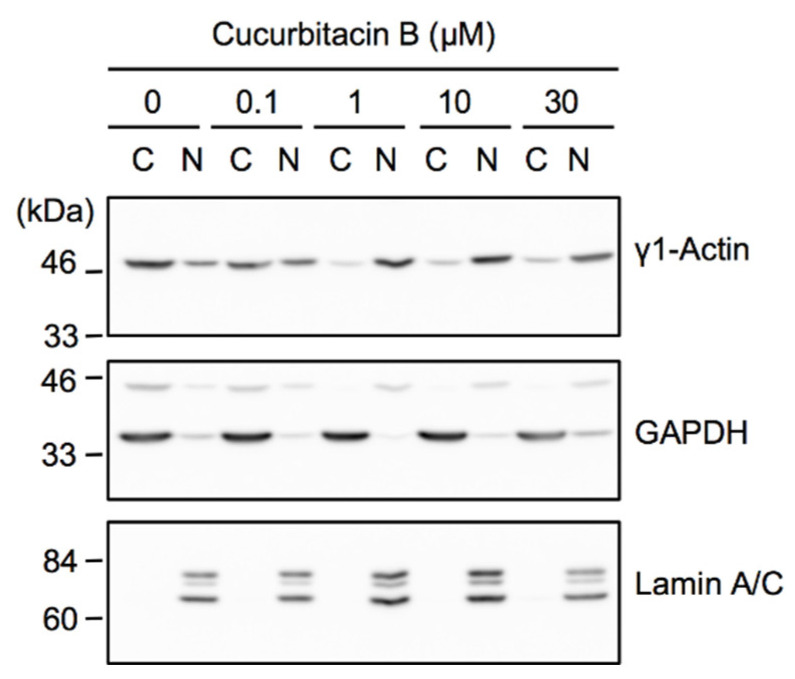
Cucurbitacin B decreased γ1-actin in cytoplasmic fractions. A549 cells were treated with the indicated concentrations of cucurbitacin B for 1 h, followed by the preparation of cytoplasmic (C) and nuclear fractions (N). Blots are representative of two independent experiments.

Cucurbitacins have been reported to induce the aggregation of actin, which decreases the globular actin (G-actin) pool, in Triton X-100-soluble fractions [42,43]. To clarify whether cucurbitacin B affected the actin cytoskeleton in A549 cells, the amount of γ1-actin in cytoplasmic fractions, which were collected as Triton X-100-soluble fractions, was analyzed by Western blotting. GAPDH, but not lamin A/C, was present in the cytoplasmic fraction, while lamin A/C was only detected in the nuclear fraction (Figure 9). Cucurbitacin B decreased γ1-actin in the cytoplasmic fraction (Figure 9). These results suggest that cucurbitacin B affected the actin cytoskeleton and decreased the G-actin pool in A549 cells.

**Figure 10:**
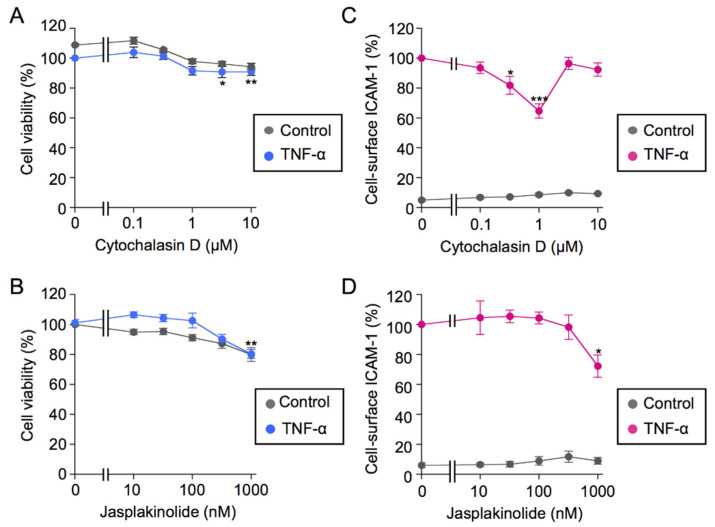
Cytochalasin D and jasplakinolide only partially inhibited TNF-α-induced cell-surface ICAM-1 expression. (**A**–**D**) A549 cells were pretreated with serial dilutions of cytochalasin D (**A**,**C**) or jasplakinolide (**B**,**D**) for 1 h and were then treated with (blue circles in **A**,**B** and red circles in **C**,**D**) or without (gray circles) TNF-α (2.5 ng/mL) in the presence of cytochalasin D or jasplakinolide at the indicated final concentrations for 6 h. Cell viability (%) (**A**,**B**) and cell-surface ICAM-1 (%) (**C**,**D**) are shown as the mean ± S.E. of three independent experiments. * *p* < 0.05, ** *p* < 0.01, and *** *p* < 0.001.

We also investigated the effects of small-molecule actin-modulating agents on TNF-α-induced ICAM-1 expression. Cytochalasin D has been shown to inhibit actin polymerization, while jasplakinolide stabilizes the actin cytoskeleton [44]. In the present study, cytochalasin D at concentrations higher than 3 µM only slightly decreased the viability of A549 cells (Figure 10A), whereas cytochalasin D at a narrow range of 0.3 or 1 µM down-regulated TNF-α-induced ICAM-1 expression by ~40% (Figure 10C). Jasplakinolide at 1 µM decreased cell viability by ~20% (Figure 10B), and down-regulated TNF-α-induced ICAM-1 expression by approximately 30% (Figure 10D). These results indicate that cytochalasin D and jasplakinolide only partially down-regulated TNF-α-induced ICAM-1 expression.

## 3. Discussion

The present results demonstrated that cucurbitacin B down-regulated TNF-α-induced ICAM-1 expression and the NF-κB signaling pathway in human lung adenocarcinoma A549 cells. We also showed that cucurbitacin B inhibited IL-1α-induced ICAM-1 expression in A549 cells. Previous studies reported that cucurbitacins suppressed the NF-κB pathway activated either constitutively or by various stimuli, including TNF-α, lipopolysaccharide, and phorbol ester plus ionomycin [19,20,21,22,23,24,25,26,27,28,29,30,31,32,33,34]. Different sets of receptors and adaptor proteins are involved in NF-κB signaling pathways, which converge to induce the activation of IκB kinase [3,9]. To the best of our knowledge, the target proteins of cucurbitacins in the NF-κB signaling pathway have not yet been clearly identified.

A previous study demonstrated that cucurbitacins B, E and I suppressed LPS-induced nuclear RelA translocation in mouse microglia [27]. Cucurbitacin E inhibited TNF-α-induced nuclear RelA translocation in human synoviocyte MH7A cells [26] and suppressed LPS-induced nuclear RelA translocation in mouse RAW264.7 cells without affecting the phosphorylation of IκBα [23]. In contrast to these findings, another study demonstrated that cucurbitacin B did not affect TNF-α-induced nuclear RelA translocation but inhibited its transcriptional activity in human cervical carcinoma HeLa cells [21]. Based on these findings, cucurbitacins appear to inhibit multiple steps in the NF-κB signaling pathway in a cell context-dependent manner. The present results showed that cucurbitacin B inhibited TNF-α-induced IκBα phosphorylation and selectively down-regulated the expression of TNF-R1 without affecting three adaptor proteins. Therefore, the inhibition of the TNF-α-induced NF-κB signaling pathway by cucurbitacin B appears to be at least partly attributed to the down-regulation of TNF-R1 in the initial step.

Glutathione, *N*-acetyl-L-cysteine, and, to a lesser extent, L-cysteine attenuated the inhibitory effects of cucurbitacin B. These results confirmed that the *α,β*-unsaturated carbonyl moiety of cucurbitacin B is critical, which is consistent with previous findings showing that *N*-acetyl-L-cysteine canceled the biological effects of cucurbitacin B in A549 cells [45]. Compounds possessing *α,β*-unsaturated carbonyl moieties have been reported to inhibit the NF-κB signaling pathway by targeting the Cys179 of IκB kinase β and Cys38 of the NF-κB subunit RelA [35,46]. We previously showed that eudesmane-type sesquiterpene lactones containing *α,β*-unsaturated carbonyl moieties, such as an α-methylene-γ-lactone or α-bromo ketone group, targeted multiple steps in the NF-κB signaling pathway induced by TNF-α and IL-1α [41]. In addition to the down-regulated expression of TNF-R1, IκB kinase β and the NF-κB subunit RelA are potential candidate targets for cucurbitacin B in the common NF-κB signaling pathway activated by TNF-α and IL-1α.

We previously reported that allantopyrone A, possessing two *α,β*-unsaturated carbonyl moieties, binds and crosslinks multiple proteins, including TNF-R1, which blocks the TNF-α-induced NF-κB pathway [47]. Allantopyrone A directly bound to TNF-R1 because it reduced the reactivity of the anti-TNF-R1 antibody to an epitope containing a cysteine residue in the extracellular CRD [47]. Western blotting using different antibodies recognizing TNF-R1 excluded the possibility that cucurbitacin B bound to TNF-R1 in a similar manner to allantopyrone A. We recently reported that isopanduratin A, a flavonoid possessing an *α,β*-unsaturated carbonyl moiety, down-regulated the expression of TNF-R1 by ectodomain shedding via the extracellular signal-regulated kinase (ERK)-dependent activation of TACE and the inhibition of de novo translation via eIF2α phosphorylation [48]. Translation inhibitors regulate the expression of TNF-R1 by reducing its de novo synthesis and by inducing its ectodomain shedding via the activation of ERK and/or p38 MAP kinase [49]. The TACE inhibitor TAPI-2 did not reverse the amount of TNF-R1 in isopanduratin A-treated A549 cells, which was the combined effect of enhanced ecodomain shedding and inhibited translation [48]. In contrast to isopanduration A, TAPI-2 suppressed reductions in TNF-R1 in cucurbitacin B-treated A549 cells. Based on the present results, we speculate that cucurbitacin B induces the TACE-dependent cleavage of TNF-R1 and then promotes the prompt degradation of intracellular TNF-R1 fragments. Further analyses are required to clarify the regulation of intracellular TNF-R1 levels by translation, ectodomain shedding, lysosomal degradation, and/or proteasomal degradation as well as the molecular mechanisms by which cucurbitacin B down-regulates TNF-R1.

Cucurbitacins have been reported to induce the aggregation of actin, which reduces the G-actin pool [42,43]. They have also been shown to covalently bind to cofilin1 via Cys39 and F-actin via Cys257 [50,51]. Cofilins cleave actin filaments and promote depolymerization, which is inactivated by phosphorylation [52]. Cucurbitacins were previously found to inhibit cofilin phosphorylation and promote actin aggregation, which was accompanied by a reduction in the G-actin pool [53,54]. Cucurbitacin B affected the actin cytoskeleton in A549 cells, as evidenced by a reduction in the G-actin pool. In contrast to cucurbitacin B, the actin-modulating agents cytochalasin D and jasplakinolide exerted only partial inhibitory effects on TNF-α-induced ICAM-1 expression in A549 cells. These results suggest that the effects of cucurbitacin B on the actin cytoskeleton are not the main mechanism by which it down-regulates TNF-α-induced ICAM-1 expression and inhibits the NF-κB signaling pathway.

In the present study, we demonstrated that cucurbitacin B down-regulated the expression of TNF-R1 and inhibited the common NF-κB signaling pathway induced by TNF-α and IL-1α. In addition, we showed that cucurbitacin B decreased γ1-actin in the cytoplasmic fraction. These results revealed that cucurbitacin B has multiple cellular targets. Consistent with this notion, cucurbitacin B has been shown to have many molecular targets in cancer signaling pathways [18]. The combined effects of cucurbitacin B targeting multiple steps in the NF-κB pathway may contribute to stronger anti-inflammatory activity. However, higher selectivity is preferred to reduce side effects and develop therapeutic drugs. Further studies are needed to identify the multiple cellular targets of cucurbitacin B in A549 cells and clarify their modes of action in the NF-κB signaling pathway induced by TNF-α and IL-1α.

## 4. Materials and Methods

### 4.1. Cells

Human lung adenocarcinoma A549 cells (JCRB0076, National Institutes of Biomedical Innovation, Health and Nutrition JCRB Cell Bank, Osaka, Japan) were cultured in RPMI 1640 medium (Thermo Fisher Scientific, Gland Island, NY, USA) supplemented with heat-inactivated fetal calf serum (Sigma-Aldrich, St. Louis, MO, USA) and a penicillin-streptomycin antibiotic mixture (Nacalai Tesque, Kyoto, Japan).

### 4.2. Reagents

Cucurbitacin B (Tokyo Chemical Industries Co., Ltd., Tokyo, Japan), bafilomycin A_1_ (Cayman Chemical Company, Ann Arbor, MI, USA), MG-132 (Peptide Institute, Osaka, Japan), TAPI-2 (Peptide Institute), *N*-acetyl-L-cysteine (Nacalai Tesque), glutathione (FUJIFILM Wako Pure Chemical Corporation, Osaka, Japan), L-cysteine (Nacalai Tesque), ascorbic acid (Nacalai Tesque), Trolox (FUJIFILM Wako Pure Chemical Corporation), cytochalasin D (Cayman Chemical Company), and jasplakinolide (Nacalai Tesque) were purchased for the present study. Human recombinant TNF-α was provided by Dainippon Pharmaceutical Co., Ltd. (Osaka, Japan).

### 4.3. Plasmids

A pCR3 expression vector encoding C-terminal FLAG-tagged human TNF-R1 was previously described [47]. A pGL4.22[luc2CP/Puro] vector containing the ICAM-1 promoter (−1604 to +40) was previously described [55]. A pCR3 expression vector encoding CMV promoter-driven *Renilla* luciferase was previously described [56].

### 4.4. Antibodies

Primary antibodies for γ1-actin (2F3; FUJIFILM Wako Pure Chemical Corporation), FLAG (1E6; FUJIFILM Wako Pure Chemical Corporation), ICAM-1 (15.2; Leinco Technologies, Inc., St. Louis, MO, USA), RelA (F-6; Santa Cruz Biotechnology, Dallas, TX, USA), lamin A/C (E-1; Santa Cruz Biotechnology), GAPDH (6C5; Santa Cruz Biotechnology), IκBα (25/IkBa/MAD-3; BD Biosciences, San Jose, CA, USA), phospho-IκBα (Ser32/36) (5A5; Cell Signaling Technology, Danvers, MA, USA), TNF-R1 (H-5; Santa Cruz Biotechnology), TNF-R1 (C25C1; Cell Signaling Technology), RIPK1 (38/RIP; BD Biosciences), TRADD (37/TRADD; BD Biosciences), and TRAF2 (F-2; Santa Cruz Biotechnology) were used. A peroxidase-conjugated anti-mouse IgG(H + L) antibody (Jackson ImmunoResearch Laboratories, West Grove, PA, USA) and peroxidase-conjugated anti-rabbit IgG(H + L) antibody (Jackson ImmunoResearch Laboratories) were used as secondary antibodies.

### 4.5. Cell Viability Assay

Cell viability was evaluated by the MTT assay, which measured mitochondrial MTT-reducing activity. A549 cells were incubated with MTT at a concentration of 500 µg/mL for the last 2 h. Formazan was extracted in the presence of 5% SDS overnight. Absorbance at 570 nm was measured by the iMark microplate reader (Bio-Rad Laboratories, Hercules, CA, USA). Cell viability (%) was calculated as (experimental absorbance—background absorbance without cells)/(control absorbance—background absorbance without cells) × 100.

### 4.6. Cell-ELISA

Cell-ELISA was performed as previously described [57]. A549 cells were washed with PBS and fixed with 1% paraformaldehyde–phosphate-buffered saline (PBS), followed by blocking with 1% bovine serum albumin–PBS overnight. Fixed cells were serially incubated with the mouse anti-ICAM-1 antibody (15.2) and peroxidase-conjugated anti-mouse IgG(H + L) antibody. After the colorimetric reaction with *o*-phenylenediamine dihydrochloride and hydrogen peroxide, absorbance at 450 nm was measured by the iMark microplate reader. ICAM-1 protein (%) was calculated as (experimental absorbance—background absorbance without cells)/(control absorbance with TNF-α or IL-1α stimulation—background absorbance without cells) × 100.

### 4.7. Flow Cytometry

Flow cytometry was performed as previously described [58]. A549 cells were stained either with the anti-ICAM-1 antibody (15.2) or an isotype control antibody (MOPC-21; BioLegend, San Diego, CA, USA) at a concentration of 1 µg/mL, followed by staining with a phycoerythrin-labeled anti-mouse IgG antibody (Jackson ImmunoResearch) at a concentration of 5 µg/mL. Fluorescent intensity was assessed by FACSCalibur (BD Biosciences). Histograms were analyzed using FlowJo software version 8.5.1 (Tomy Digital Biology, Tokyo, Japan).

### 4.8. Quantitative PCR

Total RNA was extracted with Sepasol^®^-RNA I Super G (Nacalai Tesque) and converted to cDNA by ReverTra Ace^®^ (TOYOBO, Osaka, Japan) and oligo (dT)_20_ primers. cDNA was used as a template to evaluate the expression of ICAM-1 mRNA, β-actin mRNA, TNF-R1 mRNA, and GAPDH mRNA by quantitative PCR using TB Green^®^ Premix Ex Taq^TM^ II (Tli RNase H Plus) (Takara Bio, Kusatsu, Japan) and Thermal Cycler Dice^®^ Real Time System Lite (Takara Bio). The primer pairs used were previously described: human ICAM-1 (148 bp) [59],human β-actin (143 bp) [60], human TNF-R1 (243 bp) [61], and human GAPDH (113 bp) [62].

### 4.9. Luciferase Reporter Assay

A549 cells were transfected with a pGL4.22[luc2CP/Puro] vector encoding an ICAM-1 promoter-driven firefly luciferase reporter and a pCR3 expression vector encoding a CMV promoter-driven *Renilla* luciferase reporter by HilyMax Transfection Reagent (Dojindo Laboratories, Kumamoto, Japan). Cell lysates were prepared and used for the luciferase assay as previously described [56]. Relative light units were measured by Lumitester C-110 (Kikkoman Biochemifa, Tokyo, Japan).

### 4.10. Preparation of Cell Lysates

A549 cells were treated with Triton X-100 lysis buffer (1% Triton X-100, 50 mM Tris-HCl (pH 7.4), 2 mM DTT, and 2 mM sodium vanadate) supplemented with cOmplete^TM^ Protease Inhibitor Cocktail (Sigma-Aldrich) on ice for 15 min. After centrifugation (15,300× *g*, 5 min), supernatants were collected as cytoplasmic fractions. Precipitates were further treated with sonication, and centrifuged (15,300× *g*, 5 min) to remove insoluble materials. Supernatants were collected as nuclear fractions.

### 4.11. Western Blotting

Western blotting was performed as previously described [63]. Proteins were separated by SDS-PAGE and transferred to nitrocellulose membranes. Membranes were then serially incubated with primary antibodies and peroxidase-conjugated secondary antibodies. Protein bands were acquired by Amersham Imager 680 (GE Healthcare Japan, Tokyo, Japan) and analyzed by ImageQuant TL software toolbox version 7.0 (GE Healthcare Japan).

### 4.12. Statistical Analysis

Statistical analyses were performed using a one-way ANOVA and Tukey’s test by KaleidaGraph software version 4.5.1 (Hulinks, Tokyo, Japan).

## 5. Conclusions

The present results demonstrated that cucurbitacin B down-regulated the expression of TNF-R1 and inhibited the TNF-α-dependent NF-κB signaling pathway in A549 cells. TNF-α is produced by activated macrophages and other types of cells, while TNF-R1 is ubiquitously expressed in various cells. TNF-α-induced NF-κB-dependent gene expression plays an essential role in inflammatory and immune responses. The acute and chronic activation of the NF-κB signaling pathway has been implicated in the pathogenesis of inflammatory diseases. Natural products, such as cucurbitacins, that target the TNF-α-induced NF-κB pathway have potential as anti-inflammatory agents. Further studies are needed to elucidate the mechanisms of action of cucurbitacins on the NF-κB signaling pathway and develop more selective anti-inflammatory agents. While the present study focused on the cellular response of non-immune cells stimulated with inflammatory cytokines, macrophages are involved in detrimental inflammatory diseases by producing excess inflammatory cytokines, such as TNF-α. Future research is warranted to investigate the effects of cucurbitacin B on primary macrophages and a TNF-α-induced inflammation in vivo model.

## Data Availability

The data presented in the present study are available upon request from the corresponding author.

## Supplementary Materials

The following supporting information can be downloaded at: https://www.mdpi.com/article/10.3390/ijms23137130/s1.
