# Supplementary material for: Cucurbitacin B Down-Regulates TNF Receptor 1 Expression and Inhibits the TNF-α-Dependent Nuclear Factor κB Signaling Pathway in Human Lung Adenocarcinoma A549 Cells
Source: Int J Mol Sci. 2022 Jun 27;23(13):7130. doi: 10.3390/ijms23137130 (PMC9267118; doi:10.3390/ijms23137130)
Supplement: Supplementary file 1 [file ijms-23-07130-s001.zip › ijms-1681161-supplementary.pdf]

Supplementary Materials

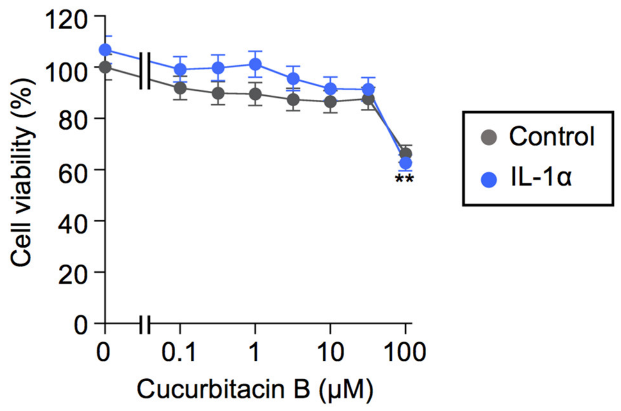

Figure S1. Cucurbitacin B did not markedly affect cell viability at concentrations up to 30 μM. A549 cells were pretreated with serial dilutions of cucurbitacin B for 1 h, and were then treated with (blue circles) or without (gray circles) IL-1α (0.25 ng/mL) in the presence of cucurbitacin B at the indicated final concentrations for 6 h. Cell viability (%) is shown as the mean ± S.E. of three independent experiments. $^{**}P < 0.01$.

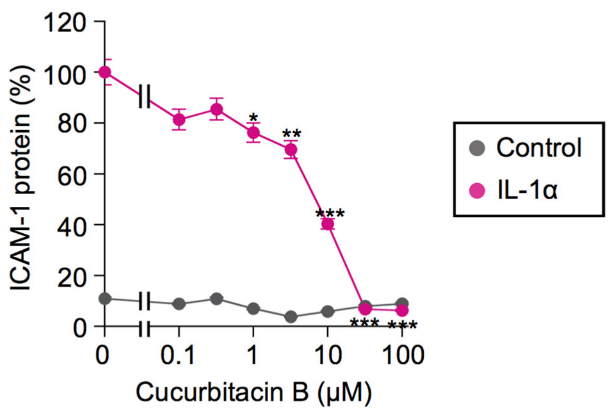

Figure S2. Cucurbitacin B inhibited the IL-1α-induced expression of ICAM-1 protein. A549 cells were pretreated with serial dilutions of cucurbitacin B for 1 h, and were then treated with (red circles) or without (gray circles) IL-1α (0.25 ng/mL) in the presence of cucurbitacin B at the indicated final concentrations for 6 h. ICAM-1 protein (%) is shown as the mean ± S.E. of three independent experiments. $^{*}P < 0.05$, $^{**}P < 0.01$, and $^{***}P < 0.001$.

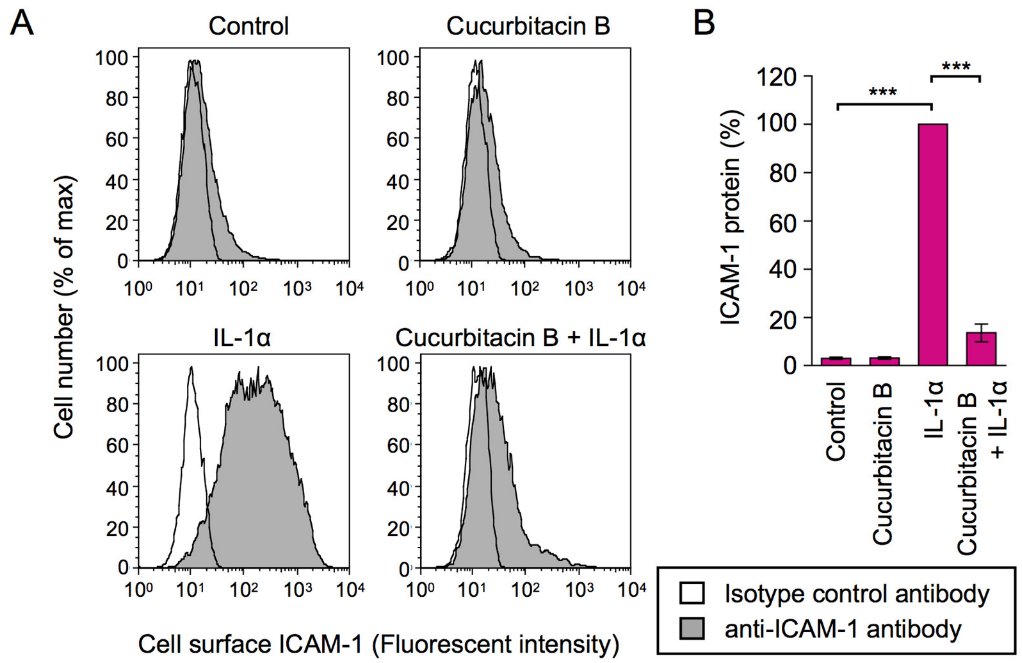

Figure S3. Cucurbitacin B inhibited IL-1α-induced cell-surface ICAM-1 expression. (**A** and **B**) A549 cells were incubated with cucurbitacin B for 1 h, and were then treated with or without IL-1α (0.25 ng/mL) in the presence or absence of cucurbitacin B (30 μM) for 6 h. Histograms are representative of three independent experiments (**A**). Cell-surface ICAM-1(%) is shown as the mean ± S.E. of three independent experiments (**B**). $^{***}P < 0.001$.

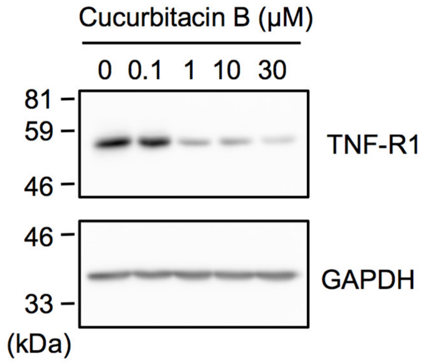

Figure S4. Cucurbitacin B down-regulated the expression of TNF-R1. A549 cells were treated with or without cucurbitacin B at the indicated concentrations for 1 h, followed by the preparation of cytoplasmic fractions. TNF-R1 was detected by the anti-TNF-R1 antibody H5, which recognized an epitope in the CRD. Blots are representative of two independent experiments.

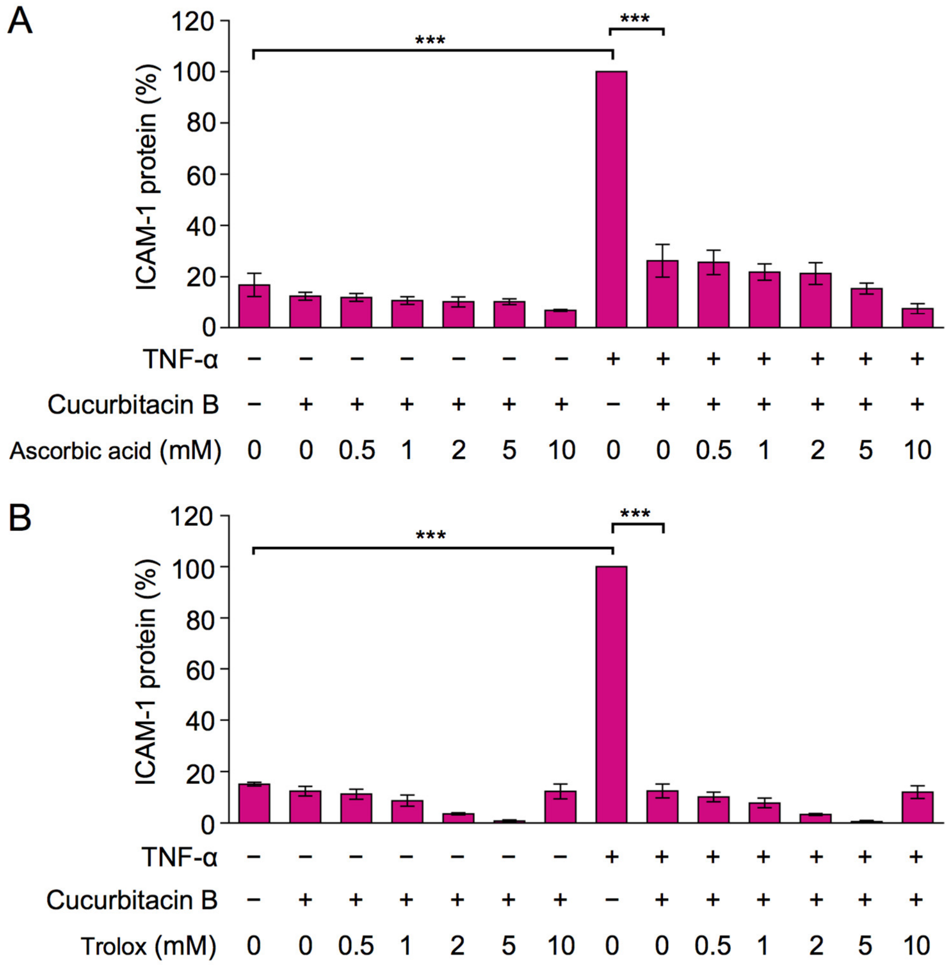

Figure S5. The inhibitory effect of cucurbitacin B was not attenuated by ascorbic acid or Trolox. (**A** and **B**) A549 cells were pretreated with serial dilutions of ascorbic acid (**A**) and Trolox (**B**) for 1 h, then with (+) or without (−) cucurbitacin B (10 µM) for 1 h, and then with (+) or without (−) TNF-α (2.5 ng/mL) for 6 h in the presence of absence of compounds at the indicated final concentrations. Cell-surface ICAM-1 (%) is shown as the mean ± S.E. of three independent experiments. $^{***}P < 0.001$.
